# Editorial: Artificial intelligence in assisted reproductive treatments

**DOI:** 10.3389/frph.2025.1704386

**Published:** 2025-10-01

**Authors:** O. Tsonis, N. Khlifa

**Affiliations:** ^1^ Fertility Preservation Service, Assisted Conception Unit, Guy’s and St Thomas’ NHS Foundation Trust, London, United Kingdom; ^2^ Tunis El Manar University, Tunis, Tunisia

**Keywords:** embryo selection, assisted conception, assisted reproductive treatment (ART), artificial intelligence (AI), sperm selection

**Editorial on the Research Topic**
Artificial intelligence in assisted reproductive treatments

Artificial intelligence (AI) is increasingly shaping the future of reproductive medicine, with applications ranging from gamete analysis to embryo selection and patient-centred care. In assisted reproductive treatments (ART), where clinical decision-making has traditionally relied on human observation, subjective judgment, and trial-and-error approaches, AI offers the potential for objectivity, reproducibility, and efficiency. This Research Topic was conceived to explore the evolving role of AI in ART and to provide insights into how these technologies can support both clinicians and patients. The five contributions presented here span a spectrum of methodologies and clinical applications, reflecting both the promise and the ongoing challenges of implementing AI in reproductive healthcare.

## AI in the context of reproductive genetics

Jia et al. investigated the role of preimplantation genetic testing for aneuploidy (PGT-A) in couples carrying pericentric inversion of chromosome 9. Through a retrospective analysis of 340 couples, they compared outcomes between those who underwent PGT-A and those treated with conventional IVF/ICSI. They found that chromosome 9 did not exhibit higher embryonic aberration rates compared with other autosomes, and that cumulative live birth rates were comparable between the two groups. While PGT-A reduced the number of transfers required to achieve live birth, overall oocyte utilization was not improved, leading the authors to conclude that PGT-A is not recommended for inversion 9 carriers. This study highlights the importance of evaluating AI-driven or adjunctive technologies critically within the clinical genetics space—reminding us that not all interventions labelled as “advanced” necessarily translate into better outcomes for all patients.

## Comprehensive perspectives on Ai in ART

Kakkar et al. provided a comprehensive review of AI integration in ART. The authors mapped current applications across sperm, oocyte, and embryo assessment, as well as predictive modelling of treatment outcomes and personalization of care. Beyond the technical innovations, the review placed a welcome emphasis on ethical and regulatory frameworks. Concerns such as algorithmic bias, transparency, and the interpretability of AI models remain central barriers to clinical adoption. The recent EU AI Act is likely to shape how reproductive medicine navigates these issues. This review underscores that while the technical capacity of AI in ART is growing rapidly, sustainable clinical translation requires as much attention to governance and ethics as to performance metrics.

## Deep learning in embryo assessment

Jia et al. also contributed a scoping review cataloguing the remarkable expansion of deep learning research using time-lapse imaging of embryos. From more than 770 screened studies, 77 were included, with convolutional neural networks dominating the field. Applications ranged from predicting blastocyst formation and implantation potential to assessing chromosomal composition. However, the review identified striking gaps: most datasets were private, often limited in size, and lacked demographic and clinical diversity. Few studies reported maternal age, and nearly all originated from high-income settings, limiting generalizability. The authors call for public, multicentre datasets and for models that can integrate multimodal data, including patient characteristics, to ensure both equity and reproducibility. Their findings remind us that AI in embryo assessment, while technically advanced, risks reinforcing inequities unless data represent the populations it intends to serve.

## AI and male infertility: advances in sperm analysis

Saadat et al. explored a modified U-Net deep learning model for detecting sperm cells in video microscopy. Using the VISEM dataset, they compared several architectures and found that UNet++ with ResNet34 delivered the most robust performance, with an AUC of 0.96. Challenges remain in differentiating sperm that are closely clustered, but the study illustrates the growing utility of AI in computer-aided sperm analysis. Importantly, these advances promise to reduce human error, standardize assessments, and enhance the reproducibility of andrology diagnostics. Together with AI in embryo evaluation, developments in gamete analysis represent a significant step toward more objective and reliable laboratory practice.

## Towards clinically integrated embryo selection algorithms

Borna et al. developed *DeepEmbryo*, an AI algorithm designed for embryo selection using three static images captured at 19, 43, and 67 h post-insemination. Unlike systems dependent on continuous time-lapse imaging, DeepEmbryo works with equipment already present in most IVF laboratories. Impressively, the algorithm achieved 75% accuracy in predicting pregnancy outcomes, outperforming both single-image models and a panel of experienced embryologists. Segmentation steps improved input quality, and the model consistently exceeded human performance in embryo evaluation. By aligning with existing workflows, DeepEmbryo exemplifies how AI can be integrated into routine practice without imposing prohibitive infrastructural demands—a critical consideration for equitable adoption across diverse clinical settings.

## Broader implications and future directions

Taken together, the five contributions highlight both the breadth of AI applications in ART and the complexity of their translation into practice. From genetic testing to gamete and embryo evaluation, these studies showcase the capacity of AI to enhance precision and reproducibility. At the same time, they illuminate recurrent challenges: limited datasets, lack of transparency, uneven global representation, and the need for careful regulatory and ethical frameworks.

The promise of AI in ART lies not only in incremental improvements to laboratory performance but also in the potential to reshape how we conceptualize care—by reducing variability, supporting equitable access, and enabling more personalized treatment journeys. For this vision to be realized, future research must prioritize multicentre collaborations, open data initiatives, explainable AI models, and careful attention to inclusivity and patient trust.

## Conclusion

This Research Topic illustrates the transformative potential of AI in assisted reproduction, while reminding us that technology alone cannot solve the complexities of infertility. True innovation requires the integration of robust science, ethical responsibility, and patient-centred care. As these five studies demonstrate, AI can serve as both a mirror reflecting our current limitations and a lens focusing our collective aspirations for the future of reproductive medicine.

